# The Developing World Urgently Needs Phages to Combat Pathogenic Bacteria

**DOI:** 10.3389/fmicb.2016.00882

**Published:** 2016-06-08

**Authors:** Tobi E. Nagel, Benjamin K. Chan, Daniel De Vos, Ayman El-Shibiny, Erastus K. Kang'ethe, Angela Makumi, Jean-Paul Pirnay

**Affiliations:** ^1^Phages for Global HealthOakland, CA, USA; ^2^Department of Ecology and Evolutionary Biology, Yale UniversityNew Haven, CT, USA; ^3^Laboratory for Molecular and Cellular Technology, Burn Wound Center, Queen Astrid Military HospitalBrussels, Belgium; ^4^Faculty of Medicine, Catholic University of BukavuBukavu, Democratic Republic of Congo; ^5^Biomedical Sciences, University of Science and Technology, Zewail City of Science and TechnologyGiza, Egypt; ^6^Department of Public Health Pharmacology and Toxicology, University of NairobiNairobi, Kenya; ^7^Department of Microbial and Molecular Systems, KU LeuvenLeuven, Belgium; ^8^Center for Microbiology Research, Kenya Medical Research InstituteNairobi, Kenya

**Keywords:** bacteriophage, phage, antibiotic, antimicrobial, resistance, infectious disease, developing countries

With the growing global antimicrobial resistance crisis, there is a critical need for alternatives to conventional antibiotics, especially in developing countries. Virulent bacteriophages (phages) represent a viable antibacterial technology that could be particularly beneficial, since phages are active against antimicrobial-resistant bacteria, easy to isolate from contaminated environments, and relatively inexpensive to produce. We discuss here examples of infectious diseases that significantly affect developing countries, phage applications that could be especially impactful in those settings, and special considerations for implementing phages in the developing world.

## Developing countries are disproportionately impacted by infectious diseases

Bacterial infections cause more loss of life and health in developing nations than in wealthy ones. As an example, infection with the bacteria *Campylobacter* is associated with an average case fatality rate of ~0.1% in developed countries, but in Kenya reportedly 8.8% of infected individuals die, with most of those deaths occurring in children (O'Reilly et al., 2012; The Global View of Campylobacteriosis, 2013). A 2014 report commissioned by the UK Prime Minister predicts that by the year 2050, almost 10 million people will die from antibiotic-resistant infections annually: roughly 4.73 million in Asia and 4.15 million in Africa, in contrast to 0.39 and 0.32 million in Europe and the US, respectively (Antimicrobial Resistance: Tackling a Crisis for the Health and Wealth of Nations, 2014). These numbers are actually underestimates, since they are based on data available for only three of the seven bacteria that the World Health Organization has deemed concerns.

Developing nations are also less able to prevent infections. Countries categorized as lower middle income countries (LMIC)—defined as those with less than $4125 gross national income *per capita*—are more likely to lack clean water and have sanitation and hygiene problems. Not only are greater numbers of people affected by bacterial pathogens in LMIC, infected individuals are often more vulnerable. Malnourished, immunocompromised, and HIV-positive patients have more severe illnesses and greater risks. Given the public health and antibiotic resistance problems in the developing world, alternative treatment modalities are urgently needed. Here, we describe how phages are particularly appropriate for LMIC.

## Phages are well-suited for applications in developing countries

For over 100 years, virulent phages have been used in the former Soviet Union as alternatives to and alongside traditional antibiotics (Abedon et al., 2011). Therapeutic phages are naturally occurring viruses that can be selected to kill only specific bacterial species or strains while leaving other, helpful bacteria and mammalian cells unharmed. Thus, unlike broad-spectrum antibiotics, they spare the commensal microbiota (e.g., in the gut)—a characteristic particularly important for malnourished and immunocompromised individuals. In addition, they can be effective against antibiotic-resistant bacteria. Phages can be easily isolated from environments enriched in targeted bacteria, such as hospital waste or sewage water, with tools readily available to people in LMIC. In principle, phage products can also be developed faster and more cost-effectively than conventional drugs, and they can be dry powder formulated so that they require no refrigeration (Semler et al., 2012).

## Current and potential uses of phages

Phage applications span a broad range, from food systems to animal husbandry and clinical therapies. Generally, only lytic phages are considered suitable for biocontrol or therapeutic applications. Unlike temperate phages, lytic phages obligately lyse bacteria and do not mediate the exchange of virulence or antibiotic resistant determinants between bacteria.

### Food decontamination

Methodologies that address foodborne diseases are particularly important for LMIC: many developing countries lack reliable refrigeration and good hygiene practices, providing ample opportunities for bacteria to grow. In Western countries, several phage products are currently approved for the control of food pathogens, including *Listeria monocytogenes, Salmonella*, and *Escherichia coli* (Naanwaab et al., 2014). They are used as sprays to decontaminate fruits, vegetables, cheese, fish, poultry, and meats. If such phage products were available in developing countries, their benefits could be even greater. For example, given the scarcity of dependable refrigeration in many LMIC, meat products are ideally sold within 24 h of slaughter. Thus, any antibacterial product that could increase the shelf life by even 1 day could have significant impacts not only on public health, but also on profitability for meat sellers.

### Veterinary applications

Phages could also be employed to decrease bacterial loads in animals before they reach slaughterhouses. For instance, *Campylobacter* is resident in the intestinal tracts of up to ~75% of poultry in many countries (Coker et al., 2002). While it causes little harm to chickens, it is the leading bacterial cause of gastroenteritis in humans worldwide. Data from Egypt shows that in households with backyard poultry that are contaminated with *Campylobacter*, children are almost four-fold more likely to be infected with the bacteria (El-Tras et al., 2015). Numerous studies have demonstrated that adding *Campylobacter* phages to chicken feed can decrease bacterial levels in experimental bird models by several orders of magnitude, presenting an attractive biocontrol strategy (Connerton et al., 2011). However, any applied phages would need to be active against the range of bacterial strains present in poultry flocks, and the environmental impacts of large-scale phage application should be studied in greater depth (Marotta et al., 2015). If not managed properly, bacteria could develop broader resistance to phages. Multiple approaches can be employed to minimize such resistance, including adapting the phages *in vitro*, utilizing phage cocktails, and switching to different phages at regular intervals (Chan et al., 2013). Phage preparations, especially for biocontrol applications, will likely need to be tailor-made for specific settings and will require periodic updates to address pathogen diversity as well as evolutionary changes in bacterial populations (Hagens and Loessner, 2010). Ideally, banks containing different, well-characterized phages should be set up and regularly updated with new phages.

Major economic markets in developing countries could also benefit from phages. A prime example is the dairy industry, in which bovine mastitis (inflammation of the mammary glands) is the leading cattle disease worldwide (Dias et al., 2013). In India, the largest producer of milk globally, bovine mastitis decreases annual milk yields by up to 70%, with financial losses of over $1 billion each year (Sharma et al., 2012; Padhy et al., 2015). Since bovine mastitis is mainly caused by bacteria (*Staphylococcus* and *Streptococcus*) where antimicrobial resistance is increasing, alternatives to antibiotic treatment are urgently needed. Bovine infections also affect human health: low levels of contamination can be difficult to detect, often leaving bacteria to be propagated and transferred to humans through milk products (Hameed et al., 2006; Wareth et al., 2014). By decreasing such infections in dairy cattle, phages could potentially contribute to both the financial and medical health of populations in LMIC.

### Human treatment

Over the past century, phages have been administered in some regions directly to people, either as prophylaxis or to treat active infections (Abedon et al., 2011). In the 1960s, phages were tested for the prevention of seasonal *Shigella* infections in the country of Georgia. More than 30,000 children received *Shigella* phages or a placebo, and there was a statistically significant reduction in the *Shigella* infection rate in the phage-treated group (Kutter et al., 2010). Estimates indicate that *Shigella* species infect 164.7 million people annually—with 163.2 million of those cases occurring in developing countries, resulting in 1.1 million deaths each year (Kotloff et al., 1999). Since *Shigella* strains now exhibit worrying rates of resistance to antibiotics, even to third generation cephalosporins, there is significant motivation to explore using *Shigella* phages in LMIC.

In the 1920s and 1930s, several studies were conducted in India testing the efficacy of orally delivered cholera phages (Summers, 1993). In one study series involving more than 800 patients, there were consistently fewer deaths in the patients who received phages. Another study tested the ability of phages to control cholera outbreaks in hundreds of villages. Two comparable (in terms of yearly cholera outbreak patterns) yet geographically separate village regions were monitored, with one of the regions receiving phage treatment and the other not. In the treated region there were no epidemics during the 6-year course of the study, whereas in the untreated region there were seasonal epidemics in the first 3 years. After that, the government ordered phage treatment in both regions, with almost no cholera cases reported during the following 3 years. The discovery and early promise of antibiotics in the 1940s eventually led to the abandonment of phage therapies such as these. Now, given the problems of antimicrobial resistance, it is appropriate to give phages another look.

While historical phage studies were not always conducted according to the rigors of modern clinical trials, they do strongly suggest that phages could be useful in outbreak settings, such as in the Haiti cholera epidemic of 2010, which affected more than 700,000 people. In such situations, phages could help fill gaps that are not fully addressed by current vaccine and hygiene strategies. For example, phage treatment could be particularly helpful for non-vaccinated populations, since phages could work in parallel with vaccines, beginning to kill intestinal bacteria within hours, while vaccines take weeks to provide full immunological protection. Phages should also be beneficial to immunocompromised individuals, whose immune systems often are not fully responsive to vaccines. And while vaccines require refrigeration, lyophilized phages can be stored at room temperatures (Semler et al., 2012). Thus, phages could be an effective addition to the arsenal of tools already available to combat cholera outbreaks.

## Regulatory considerations

To fully realize the public health benefits of phages in LMIC, each country will need to establish appropriate regulatory guidelines. Unfortunately, traditional regulatory systems are not well-suited for the development of sustainable phage products. For example, most regulatory structures focus on drugs that are composed of a single chemical entity, whereas phage preparations would ideally be custom-made and updated at regular intervals to both enhance their efficacy and minimize the development of bacterial resistance. Since phages are naturally occurring entities, many experts have argued that a different type of regulatory process should be established for phages, perhaps something closer to that governing probiotics. One alternative approach currently being explored at the Queen Astrid Military Hospital in Belgium is to produce phages in compliance with a formal monograph describing the standards by which the phage quality will be judged for specific applications (e.g., pharmaceuticals, dietary supplements, or food ingredients; Merabishvili et al., 2009).

## Importance of local cultural contexts

Even though phages have a long history of being used as antibacterial agents in some regions of the world, they are a relatively new technology in most countries, especially in this modern era. As such, phages are bound to elicit fear and myths as novel biocontrol substances. To clarify any misperceptions, all potential stakeholders must engage in public dialogue—from regulatory authorities and policymakers to farmers, merchants, health care practitioners, and people who might receive treatment. Workshops with relevant stakeholders could provide information regarding the potential benefits of phages, address concerns, and gain input on how phages could be utilized suitably within a local social setting. The criteria and processes by which phages would be certified as safe should be clearly communicated, and the ways they will be regulated and monitored must be explicitly defined. Facilitation of public discussion about phages can broaden acceptance of this new class of antibacterial products.

While there is great potential for phage applications, considerable work must be done before phages can be adopted in LMIC. It will be important to better understand how phages co-evolve with their bacterial hosts, to develop processes for regularly updating phage cocktails, and to optimize phage therapy regimens. In addition, safety assessments, regulatory procedures, and cultural buy-in will all be essential. Obtaining intellectual property can be difficult, especially on naturally occurring phages, so there is also a need to create commercial incentives other than patents to encourage companies to develop new phage products. Perhaps progress in this area will be motivated by both the immense public health needs, as well as by the tremendous size of developing country emerging markets. Ultimately, phage products have the potential to transform how infectious diseases are addressed in developing countries, helping to save lives as well as build economies.

## Author contributions

TN conceived of the overall topic. TN, BC, DD, AE, EK, AM, and JP defined the contents of the manuscript and wrote the text. TN, AE, AM, and JP edited the manuscript. All authors read and approved the final manuscript.

## Funding

Publication of this manuscript was supported by grant HFM15/5 from the Belgian Royal Higher Institute for Defense.

### Conflict of interest statement

The authors declare that the research was conducted in the absence of any commercial or financial relationships that could be construed as a potential conflict of interest.

## Abbreviations

LMIC: lower middle income countries.
